# Association of serum butyrylcholinesterase with concurrent dry-weight change in maintenance hemodialysis: a retrospective longitudinal study

**DOI:** 10.1186/s12882-026-05185-5

**Published:** 2026-07-03

**Authors:** Hirosuke Nakata, Ryo Kamimatsuse, Koichi Nishiwaki

**Affiliations:** Department of Nephrology, Uji Tokushukai Medical Center, 145 Ishibashi, Makishima-cho, Uji, Kyoto, 611-0041 Japan

**Keywords:** Butyrylcholinesterase, Albumin, Dry weight, Hemodialysis, Nutrition, Volume status

## Abstract

**Background:**

Assessing nutrition-related change in maintenance hemodialysis is challenging because commonly used biomarkers such as serum albumin are influenced by inflammation and volume status. Prescribed dry weight (DW) is a clinically important treatment target, but longitudinal DW change may reflect both volume-related and nutrition-related body-mass change. Serum butyrylcholinesterase (BChE), a liver-synthesized protein, may better capture nutrition-related change in this setting. We examined whether longitudinal change in BChE was associated with concurrent change in prescribed DW and compared this association with that of albumin while accounting for volume-related change using human atrial natriuretic peptide (hANP).

**Methods:**

We conducted a single-center retrospective longitudinal observational study of adults receiving maintenance hemodialysis between September 2024 and December 2025. Monthly clinical data were used to calculate concurrent 3-month changes, expressed as log-ratios (Δlog), in BChE, albumin, hANP, and prescribed DW. The primary analysis evaluated the association between ΔlogBChE and concurrent ΔlogDW across repeated 3-month windows using linear regression with patient-level cluster-robust standard errors. Multivariable models included ΔlogBChE, ΔlogAlb, and ΔloghANP.

**Results:**

Ninety-six patients were included, contributing 1,248 concurrent 3-month windows. In multivariable longitudinal models, ΔlogBChE was independently associated with concurrent ΔlogDW (standardized β = 0.115; *P* = 0.005), whereas ΔlogAlb was not (*P* = 0.39). ΔloghANP was also independently associated with ΔlogDW (standardized β = 0.144; *P* = 0.001).

**Conclusions:**

In maintenance hemodialysis, longitudinal change in serum BChE was associated with concurrent prescribed DW change independent of albumin and hANP. These preliminary findings suggest that BChE may help contextualize prescribed DW changes as a nutrition-related biochemical signal, but prospective validation using standardized body-composition assessment is needed.

**Supplementary Information:**

The online version contains supplementary material available at https://doi.org/10.1186/s12882-026-05185-5.

## Background

Protein–energy wasting (PEW) remains a major clinical problem in patients undergoing maintenance hemodialysis and is strongly associated with adverse outcomes [1–3]. Serum albumin is widely used as a surrogate marker of nutritional status, but its interpretation is limited because albumin is strongly influenced by inflammation, fluid dilution, and hepatic acute-phase responses [4, 5].

Prescribed dry weight (DW) is a central treatment target in maintenance hemodialysis, but changes in DW may reflect both extracellular volume adjustment and changes in underlying body mass [6–8]. Thus, DW change alone does not necessarily indicate whether the change is primarily volume-related or accompanied by nutrition-related body-mass change.

Serum butyrylcholinesterase (BChE) is synthesized in the liver and reflects hepatic synthetic capacity and nutritional reserves [9]. Previous studies have reported associations between BChE and nutritional status in chronic illness, including dialysis populations [10]. Because albumin is affected by inflammation and dilution, BChE may provide a different nutrition-related biochemical signal, although BChE is also influenced by inflammation [11]. Longitudinal data examining whether BChE is associated with clinically relevant changes in prescribed DW remain limited.

Human atrial natriuretic peptide (hANP) is used in Japan as a pragmatic volume-related marker in hemodialysis practice [12, 13], although natriuretic peptides also reflect cardiac structure and function [14, 15]. We hypothesized that longitudinal changes in serum BChE would be associated with concurrent changes in prescribed DW in maintenance hemodialysis, independent of albumin and the volume-related marker hANP. Therefore, this study aimed to evaluate whether concurrent 3-month changes in serum BChE were associated with concurrent changes in prescribed DW.

## Methods

### Study design and participants

This was a single-center retrospective observational study conducted at a participating institution in Japan. Adult patients receiving maintenance hemodialysis three times weekly between September 2024 and December 2025 were screened. Exclusion criteria were: (1) age < 18 years; (2) DW not stably defined during the observation period or frequent failure to reach the prescribed post-dialysis DW; (3) severe hepatic dysfunction (Child-Pugh class C) or acute liver injury likely to strongly affect BChE; (4) transfer to another facility during follow-up; and (5) missing BChE or DW data. Although BChE can be influenced by conditions such as nephrotic syndrome and hyperthyroidism, no patients had clinically apparent nephrotic syndrome or overt hyperthyroidism in this cohort.

## Dry weight definition

DW was defined as the prescribed post-dialysis target weight determined by the treating nephrologist. Prescribed DW was determined on the basis of routine clinical assessment, including intradialytic symptoms, blood pressure trends, interdialytic weight gain, edema, chest radiographic findings, and other available clinical information. hANP was available as a supportive volume-related marker in some clinical assessments but was not used according to a prespecified protocol for DW adjustment. DW could be adjusted during routine care. Because no independent gold-standard measure of “optimal” DW was available, this study evaluated associations with physician-adjusted prescribed DW trajectories rather than validated euvolemic body weight.

## Clinical and laboratory variables

Monthly laboratory measurements (BChE, albumin, C-reactive protein [CRP], and hANP), prescribed DW, and dialysis adequacy (Kt/V) were obtained as part of routine care. For longitudinal analyses, DW, BChE, albumin, hANP, and CRP were natural log-transformed; 3-month changes were calculated within individuals as ΔlogX = log(X_t) − log(X_t − 3), equivalent to log(X_t/X_t − 3). For baseline cross-sectional analyses, prescribed DW and continuous clinical and laboratory predictors were also natural log-transformed unless otherwise specified. Concurrent ΔlogDW and changes in biomarkers were defined over the same 3-month window. Blood samples for BChE, albumin, and CRP were collected at the beginning of each month before a midweek dialysis session (predialysis). hANP was measured from a sample obtained immediately before blood return at the end of the same dialysis session.

Dialysis modality was recorded as hemodialysis (HD) or hemodiafiltration (HDF). The cardiothoracic ratio (CTR) was obtained from routine chest radiographs (expressed as %). Chest radiographs for CTR assessment were obtained routinely once monthly, typically mid-week during the week of each month. SBP and DBP were measured before the dialysis session.

### Outcomes

The primary endpoint was the association between concurrent 3-month changes in BChE (ΔlogBChE) and concurrent 3-month changes in prescribed DW (ΔlogDW). Secondary endpoints included the association between ΔlogAlb and ΔlogDW, and evaluation of ΔloghANP as a volume-related marker in multivariable models. In prespecified sensitivity analyses, we additionally adjusted for dialysis modality (HD vs. HDF) and CTR as pragmatic surrogates for treatment modality and volume/cardiac size. These analyses were intended to examine whether BChE retained an association with DW change under clinically relevant conditions in which volume-related and treatment-related factors coexist.

### Statistical analysis

Continuous variables are summarized as mean ± standard deviation or median (interquartile range) as appropriate. Associations between changes in biomarkers and changes in DW were evaluated using linear regression. Because repeated observations were available for each patient, models used cluster-robust standard errors at the patient level. Multivariable models included markers reflecting volume status (ΔloghANP) and other prespecified covariates as appropriate. In prespecified sensitivity analyses, we additionally adjusted for dialysis modality (HD vs. HDF) and CTR as pragmatic surrogates for modality and volume/cardiac size; CTR was treated as a time-varying covariate measured at the start of each 3-month window. Delivered Kt/V was likewise entered as a time-varying covariate measured at the start of each 3-month window (t − 3). Secondary analyses included baseline cross-sectional associations, and sensitivity analyses assessed the robustness of the longitudinal findings under additional covariate adjustment and alternative approaches to within-patient dependence. To assess potential multicollinearity among ΔlogBChE, ΔlogAlb, and ΔloghANP, variance inflation factors (VIFs) were calculated for the primary multivariable model. As a post hoc exploratory analysis, we examined associations between 12-month changes in serum BChE and changes in systolic blood pressure (SBP) and diastolic blood pressure (DBP) at two annual December assessments 12 months apart. Pearson and Spearman correlation coefficients were initially calculated. We then fitted linear regression models with HC3 heteroskedasticity-robust standard errors, using ΔSBP or ΔDBP as the outcome and ΔBChE, change in the number of antihypertensive tablets taken per day, and change in prescribed DW as predictors. ΔBChE was scaled per 50-U/L increase, and ΔDW was entered per 1-kg increase. As sensitivity analyses, baseline BP was additionally included, and ΔDW in kilograms was replaced with percentage change in DW. Tablet count was used as a pragmatic measure and does not represent drug class or dose-equivalent antihypertensive treatment intensity. Given the exploratory nature of these analyses, P values were not adjusted for multiple comparisons.

### Statistical approach for overlapping rolling windows

Because 3-month rolling windows overlap within individuals, observations are not independent. Therefore, our primary analyses used ordinary least squares with patient-clustered robust standard errors. As sensitivity analyses addressing within-patient dependence under alternative modeling frameworks, we additionally fitted (i) a linear mixed-effects model with a random intercept for patient and (ii) a generalized estimating equations model with an exchangeable working correlation.

Two-sided P values < 0.05 were considered statistically significant. Statistical analyses were performed using EZR (Jichi Medical University, Tochigi, Japan), which is a graphical user interface for R version 4.5.2.

## Results

### Study population and data completeness

Among 113 screened patients, 17 were excluded because DW could not be stably defined, leaving 96 patients in the analytic cohort. Monthly measurements of BChE, albumin, hANP, CRP, and prescribed DW were available for the variables used in the primary longitudinal model. The primary longitudinal analysis included 1,248 concurrent 3-month windows from 96 patients, corresponding to 13 windows per patient. Observation numbers differed slightly in fully adjusted and sensitivity analyses because of missing data for some covariates, including Kt/V and CTR. Baseline patient characteristics are summarized in Table 1.

**Table 1:** Baseline characteristics of the study cohort

Characteristic	All patients (*N* = 96)
Age, y	72.40 ± 12.10
Sex, male	62 (64.6%)
Dialysis vintage, months	67.00 [36.75, 103.30]
Diabetes mellitus	43 (44.8%)
Body mass index, kg/m²	23.60 ± 3.90
Dry weight, kg	63.20 ± 12.30
Kt/V	1.44 ± 0.26
Hemoglobin, g/dL	11.10 ± 1.40
Ferritin, ng/mL	132.00 [73.75, 281.00]
Transferrin saturation, %	27.30 ± 12.40
Butyrylcholinesterase, U/L	217.60 ± 62.50
Albumin, g/dL	3.54 ± 0.40
C-reactive protein, mg/dL	0.17 [0.07, 0.38]
hANP, pg/mL	52.00 [26.20, 87.45]
GNRI	92.80 ± 6.80

Values are presented as mean ± SD, median [IQR], or n (%), as appropriate

At baseline (month 0), BChE showed moderate correlations with serum albumin (Spearman ρ = 0.298, *P* = 0.0032) and GNRI (Spearman ρ = 0.393, *P* < 0.0001), supporting its relevance as a nutrition-related marker (Additional file 1: Table S1). Table 2 provides a cross-sectional snapshot of factors associated with baseline DW, highlighting that DW reflects both body size/nutritional reserves and volume-related conditions; these baseline findings helped contextualize subsequent longitudinal models.

**Table 2:** Cross-sectional associations of baseline log-transformed prescribed dry weight with clinical and laboratory parameters

Predictor	β (95% CI)	*P* value
BChE	0.215 (0.083, 0.348)	0.0015
Albumin	0.488 (0.214, 0.763)	< 0.001
C-reactive protein	0.020 (-0.005, 0.045)	0.12
hANP	-0.054 (-0.105, -0.003)	0.038

Values are unstandardized regression coefficients estimated using linear regression with robust standard errors. Baseline prescribed DW and continuous predictors were natural log-transformed; therefore, coefficients represent log-log associations. *N = 96.*

Outcome: log-transformed prescribed dry weight (baseline, month 0)

### Primary longitudinal analysis: concurrent 3-month changes

In the primary analysis using concurrent 3-month windows, ΔlogBChE was positively associated with ΔlogDW after accounting for volume-related changes, whereas ΔlogAlb showed weaker or non-significant associations. These findings suggest that BChE and albumin may differ in their longitudinal associations with DW change. In models that included ΔlogBChE, ΔlogAlb, and ΔloghANP, ΔlogBChE remained independently associated with ΔlogDW (standardized β = 0.115; *P* = 0.005), while ΔlogAlb was not (*P* = 0.39). ΔloghANP was also associated with ΔlogDW (standardized β = 0.144; *P* = 0.001). Detailed model outputs are provided in Table 3. Fully adjusted results are shown in Additional file 1: Table S2. A sensitivity model replacing ΔlogAlb with ΔGNRI is shown in Additional file 1: Table S3, and sensitivity analyses additionally including CTR and dialysis modality are shown in Additional file 1: Table S4. Alternative approaches to within-patient dependence (LMM/GEE) are summarized in Additional file 1: Table S5. In the fully adjusted model, delivered Kt/V was independently associated with ΔlogDW (unstandardized β = 0.009; standardized β = 0.204; *P* < 0.001), and diabetes mellitus was also independently associated with ΔlogDW (unstandardized β = -0.003; standardized β = -0.181; *P* < 0.001).

**Table 3:** Primary model: association of concurrent 3-month changes with ΔlogDW (rolling 3-month windows)

Variable	β (95% CI)	Standardized β	*P* value
ΔlogBChE (3 months)	0.050 (0.015, 0.085)	0.115	0.005
ΔlogAlb (3 months)	-0.024 (-0.079, 0.031)	-0.036	0.39
ΔloghANP (3 months)	0.011 (0.004, 0.018)	0.144	0.001

ΔlogX denotes the difference in natural log-transformed values between the start and end of each 3-month window (i.e., log[X_end/X_start])

In sensitivity analyses additionally adjusting for dialysis modality and CTR, the association between concurrent ΔlogBChE and ΔlogDW remained statistically significant (β = 0.041, 95% CI 0.005–0.077, *P* = 0.026). CTR showed a borderline association with ΔlogDW (β = -0.000453, *P* = 0.068), and dialysis modality showed a borderline association (β = 0.0057, *P* = 0.054). Detailed model outputs are provided in Additional file 1: Table S4. VIFs for ΔlogBChE, ΔlogAlb, and ΔloghANP in the primary multivariable model were low, indicating no substantial multicollinearity (Additional file 1: Table S6).

### Within-patient trajectories

Representative individual trajectories of BChE, albumin, and DW are shown in Additional file 2: Figure S1. BChE demonstrated relatively smooth within-patient changes, whereas albumin displayed greater short-term variability. DW changed in a stepwise manner consistent with clinical adjustment.

### Sensitivity analyses

To assess the robustness of inference given overlapping 3-month rolling windows, we re-estimated the primary model using a linear mixed-effects model (random intercept for patient) and a generalized estimating equations model with an exchangeable working correlation (Additional file 1: Table S5). The direction of association for ΔlogBChE and ΔloghANP was consistent across approaches, and the precision of inference for ΔlogBChE varied with the correlation assumptions.

### Hypertension-related exploratory analyses

In post hoc exploratory analyses, 12-month change in BChE was not correlated with change in SBP or DBP. After adjustment for changes in the number of antihypertensive tablets taken per day and prescribed DW, a 50-U/L increase in ΔBChE was not associated with ΔSBP (β = 2.239 mmHg; 95% CI, − 4.29 to 8.77; *P* = 0.498) or ΔDBP (β = −0.167 mmHg; 95% CI, − 3.94 to 3.60; *P* = 0.930). Additional adjustment for baseline BP did not materially change the results. Replacing ΔDW in kilograms with percentage change in DW also yielded similar findings (Additional file 1: Table S7).

## Discussion

In this longitudinal study of patients undergoing maintenance hemodialysis, increases in serum BChE were associated with concurrent increases in prescribed DW, whereas changes in albumin were not. This association persisted after accounting for hANP, a volume-related marker. These findings do not indicate that BChE determines DW or directly distinguishes tissue mass from fluid volume at the individual-patient level. Rather, BChE may provide biochemical context for interpreting prescribed DW changes as a nutrition-related signal.

Prescribed DW is a composite clinical target influenced by both extracellular volume and underlying tissue mass. Changes in hANP, a marker of atrial stretch and volume load [12–15], were independently associated with changes in DW, supporting its role as a volume-related signal. In parallel, changes in serum BChE were independently associated with changes in DW, even after accounting for the volume-related marker hANP. Although CTR is commonly used as a pragmatic surrogate of volume/cardiac size in hemodialysis practice [16], adjustment for CTR and dialysis modality did not materially alter this association.

Notably, an inverse correlation between ΔlogBChE and ΔloghANP is clinically plausible because improvements in volume status (lower hANP) may occur alongside recovery of nutritional reserves and/or reduced dilution (higher BChE). In our rolling-window dataset, concurrent ΔlogBChE and ΔloghANP were moderately inversely correlated (Spearman ρ = -0.315; *P* < 0.0001; Additional file 1: Table S8). Accordingly, ΔloghANP and ΔlogBChE can move in opposite directions while remaining jointly informative, as they preferentially reflect volume-related versus nutrition-related components of DW change. Because hANP reflects cardiac stretch whereas BChE is a liver-synthesized protein related to hepatic synthetic capacity and nutritional reserves, these markers should be interpreted as complementary rather than interchangeable. In this context, “complementary information” means biochemical context that is not obtained from the DW value alone. A decrease in prescribed DW may represent correction of volume overload, loss of nutritional body mass, or both. Conversely, stable DW may mask loss of tissue mass if extracellular volume increases. BChE would not replace DW assessment, hANP, physical examination, or body-composition measurement, but may help indicate whether DW change is accompanied by a nutrition-related biochemical change.

In response to previous concerns regarding a possible relationship between BChE and blood pressure [17, 18], we performed a post hoc exploratory analysis. Twelve-month change in BChE was not associated with changes in SBP or DBP after adjustment for changes in prescribed DW and antihypertensive tablet count. This result should be interpreted cautiously because BP was assessed at selected annual time points and antihypertensive treatment was recorded as tablet count rather than drug class or dose-equivalent intensity.

Although albumin is widely used as a nutritional marker in dialysis patients, it is strongly influenced by inflammation and fluid dilution [4, 19]. In our study, albumin was associated with DW in cross-sectional analyses but failed to be associated with longitudinal DW changes. This discrepancy highlights albumin’s role as a composite or downstream marker, rather than a reliable marker for interpreting short- to intermediate-term changes in nutritional status or body composition in routine dialysis care.

Several mechanisms may explain why BChE showed a more consistent association with DW changes than albumin. First, the circulating half-life of serum albumin is approximately 19–21 days [20], whereas BChE has a shorter half-life of approximately 12 days [9]. This difference may allow BChE to respond more promptly to short-term changes in protein-energy status. Second, albumin (approximately 66.5 kDa) is substantially smaller than the predominant tetrameric form of BChE (approximately 340 kDa) [21, 22]. Therefore, the larger molecular size of BChE may make substantial dialytic removal less likely than that of albumin. However, we did not directly measure dialytic BChE removal, and membrane characteristics, convective therapies, and protein adsorption may influence measured concentrations.

Several composite indices, such as GNRI, are used in dialysis practice to assess nutritional risk [23], but many incorporate serum albumin and therefore inherit its susceptibility to inflammation and dilution. In our cohort, baseline GNRI values suggested a substantial burden of nutritional risk, yet longitudinal interpretation remains challenging when albumin fluctuates for non-nutritional reasons. Beyond albumin, circulating markers such as prealbumin (transthyretin), transferrin, and total cholesterol have been used to reflect protein–energy status [1, 24]; however, each can be influenced by inflammation, hepatic synthesis, and dilution. Our findings support BChE as a complementary biochemical marker that may be particularly useful for repeated assessment when the clinical question is how to interpret a change in prescribed DW in the context of possible coexisting nutritional and volume-related changes.

DW reduction is already incorporated, directly or indirectly, into several nutritional assessment frameworks used in dialysis or general clinical nutrition, including SGA, MIS, GLIM, and PEW-related assessments [1, 24–27]. Therefore, the potential value of BChE is not the detection of DW change itself, but the interpretation of whether DW change is accompanied by a nutrition-related biochemical change. Future prospective studies should test whether combined changes in DW and BChE identify nutritional deterioration, body-composition change, or adverse outcomes more accurately than DW change alone.

Taken together, these findings support BChE as a complementary biochemical marker for interpreting prescribed DW trajectories, particularly when nutritional and volume-related changes may coexist. Future multicenter prospective studies with direct body-composition measures are needed to validate whether ΔlogBChE helps distinguish tissue loss from volume-driven DW adjustment in broader hemodialysis populations.

Finally, BChE may have broader clinical relevance beyond nutritional assessment. Lower serum BChE has been associated with worse survival in maintenance hemodialysis cohorts. For example, low butyrylcholinesterase activity/level predicted higher all-cause mortality and provided prognostic information alongside conventional markers such as albumin and CRP [28, 29]. Although mortality was not assessed in our study, these prior findings support the potential clinical relevance of BChE-informed interpretation of DW trajectories. A possible future application is earlier recognition of nutrition-related body-mass loss in patients whose prescribed DW is being adjusted during routine care, by integrating serial BChE changes with DW trajectories and volume-related markers.

Several limitations warrant consideration. First, we did not validate whether prescribed DW was truly optimal using an independent gold-standard method. DW is a clinically prescribed target that may be modified in response to symptoms, interdialytic weight gain, blood pressure trends, chest radiographic findings, clinician judgment, and, in some cases, supportive markers such as hANP; therefore, changes in DW do not uniquely represent changes in body composition or euvolemic body weight, and incorporation bias cannot be excluded. Thus, our analyses should be interpreted as evaluating biomarker associations with real-world physician-adjusted DW trajectories rather than with a gold-standard measure of euvolemic or nutritionally appropriate body weight. Second, although some body composition measurements were available as part of routine care, standardized BIA/BCM assessments were not performed at the same monthly frequency as the biochemical measurements and were therefore not incorporated into the primary longitudinal analysis. Accordingly, we did not have concurrent objective measures of extracellular water or body composition for each 3-month window. Future prospective studies should include standardized body composition assessment, such as ECW/TBW or ECW/ICW ratios, to validate whether BChE distinguishes tissue loss from volume-driven DW adjustment. Third, unmeasured time-varying factors—including intercurrent infection or hospitalization, dietary intake and counseling, medication changes, and dialysis-related parameters such as membrane protein loss or convective dose—may have influenced both BChE and DW trajectories. Fourth, laboratory measurements were obtained monthly and hANP was sampled post-dialysis, whereas other laboratory measurements were obtained predialysis; this difference in sampling timing may introduce variability. Fifth, BNP was not measured consistently enough for inclusion in the longitudinal analysis, and MIS was not available because this was a retrospective study and standardized monthly MIS assessments were not routinely performed. Sixth, the post hoc blood pressure analysis was based on only two annual assessments 12 months apart. Therefore, it could not evaluate true longitudinal blood pressure trajectories, short-term temporal variability, seasonal variation, or potential non-linear relationships between BChE and blood pressure. In addition, blood pressure was assessed using predialysis measurements at selected time points, and antihypertensive treatment was recorded as tablet count rather than drug class or dose-equivalent treatment intensity. Finally, this was a single-center observational study; thus, external validity may be limited, and causal inference is not possible. Accordingly, our findings should be interpreted as hypothesis-generating and require validation in larger prospective studies with direct body-composition assessment.

## Conclusions

In maintenance hemodialysis, increases in serum BChE were associated with concurrent increases in prescribed DW, independent of albumin and hANP. This finding suggests that BChE may help contextualize prescribed DW changes as a nutrition-related biochemical signal, rather than serving as a direct measure of nutritional status or optimal DW. Future prospective studies should determine whether combined changes in DW and BChE identify nutritional deterioration, body-composition change, or clinically relevant outcomes more accurately than DW change alone.

## Supplementary Information

Below is the link to the electronic supplementary material.

Supplementary Material 1

Supplementary Material 2

## Data Availability

The datasets generated and/or analyzed during the current study are not publicly available because they contain potentially identifiable clinical information but are available from the corresponding author on reasonable request, subject to institutional approval and applicable data protection requirements.

## Abbreviations

DW: Dry weight
BChE: Butyrylcholinesterase
hANP: human atrial natriuretic peptide
PEW: Protein-energy wasting
CRP: C-reactive protein
Kt/V: Dialysis adequacy index
HD: Hemodialysis
HDF: Hemodiafiltration
CTR: Cardiothoracic ratio
EZR: Easy R
GNRI: Geriatric Nutritional Risk Index
VIF: Variance inflation factor
SBP: Systolic blood pressure
DBP: Diastolic blood pressure
BP: Blood pressure
SGA: Subjective Global Assessment
MIS: Malnutrition-Inflammation Score
GLIM: Global Leadership Initiative on Malnutrition
