# Supplementary material for: Association of serum butyrylcholinesterase with concurrent dry-weight change in maintenance hemodialysis: a retrospective longitudinal study
Source: BMC Nephrol. 2026 Jul 3;27:567. doi: 10.1186/s12882-026-05185-5 (PMC13621492; doi:10.1186/s12882-026-05185-5)
Supplement: Supplementary file 1 [file 12882_2026_5185_MOESM1_ESM.docx]

**Additional file 2: Supplementary Figure S1**

**Figure S1.** Individual longitudinal trajectories of biochemical markers and dry weight.

Representative individual trajectories of (A) serum BChE, (B) serum albumin, and (C) DW over time are shown for a randomly selected subset of patients. BChE demonstrates relatively smooth within-patient changes, whereas albumin shows greater short-term variability. DW changes occur in a stepwise manner reflecting clinical adjustment.
