# Supplementary material for: Association of serum butyrylcholinesterase with concurrent dry-weight change in maintenance hemodialysis: a retrospective longitudinal study
Source: BMC Nephrol. 2026 Jul 3;27:567. doi: 10.1186/s12882-026-05185-5 (PMC13621492; doi:10.1186/s12882-026-05185-5)
Supplement: Supplementary file 2 [file 12882_2026_5185_MOESM2_ESM.docx]

**Additional file 1: Supplementary Tables S1–S8**

Table S1: Baseline correlation of BChE with albumin and GNRI

Table S2: Fully adjusted model for 3-month changes in ΔlogDW (rolling 3-month windows)

Table S3: Sensitivity model replacing ΔlogAlb with ΔGNRI (albumin excluded) for concurrent 3-month changes in ΔlogDW (rolling 3-month windows)

Table S4: Sensitivity analyses including dialysis modality and cardiothoracic ratio (CTR)

Table S5: Sensitivity analyses accounting for within-patient dependence across overlapping 3-month rolling windows

Table S6: Variance inflation factors for predictors in the primary multivariable model

Table S7: Exploratory analyses of associations between 12-month changes in serum BChE and blood pressure

Table S8: Correlation between concurrent 3-month changes in biomarkers across rolling windows

Abbreviations: BChE, butyrylcholinesterase; GNRI, Geriatric Nutritional Risk Index; DW, prescribed dry weight; hANP, human atrial natriuretic peptide; CRP, C-reactive protein; HD, hemodialysis; HDF, hemodiafiltration; CTR, cardiothoracic ratio; OLS, ordinary least squares; SE, standard error; GEE, generalized estimating equations; BP, blood pressure; SBP, systolic blood pressure; DBP, diastolic blood pressure; CI, confidence interval; VIF, variance inflation factor.

**Table S1.** Baseline correlation of BChE with albumin and GNRI

*Baseline values were defined as measurements at month 0. Spearman rank correlation coefficients are shown.*

| Variables | N (observations) | Spearman ρ | P value |
| --- | --- | --- | --- |
| BChE vs albumin | 96 | 0.298 | 0.0032 |
| BChE vs GNRI | 96 | 0.393 | <0.001 |
| BChE vs albumin  (partial, adjusted for CRP)* | 96 | 0.311 | 0.002 |
| BChE vs GNRI  (partial, adjusted for CRP)* | 96 | 0.402 | <0.001 |

**Partial correlation was approximated using rank-transformed residualization (partial Spearman).*

**Table S2.** Fully adjusted model for 3-month changes in ΔlogDW (rolling 3-month windows).

Patient-clustered robust standard errors were used. Unstandardized coefficients are reported on the original regression scale using log-transformed variables; standardized coefficients are from z-score–standardized variables. ΔlogX denotes the difference in natural log-transformed values between the start and end of each 3-month window (log[X_end/X_start]).

| **Predictor** | **Unstandardized β** | **Standardized β** | **P value** |
| --- | --- | --- | --- |
| ΔlogBChE | 0.018 | 0.096 | 0.026 |
| ΔlogAlb | -0.003 | -0.011 | 0.82 |
| ΔloghANP | 0.004 | 0.132 | 0.002 |
| ΔlogCRP | 0.000 | 0.005 | 0.87 |
| Sex | 0.000 | 0.012 | 0.82 |
| Age, years | -0.000 | -0.004 | 0.95 |
| Diabetes mellitus | -0.003 | -0.181 | <0.001 |
| Dialysis modality  (HDF vs HD) | 0.002 | 0.121 | 0.054 |
| Dialysis vintage, months | -0.000 | -0.066 | 0.19 |
| Delivered Kt/V (baseline) | 0.009 | 0.204 | <0.001 |

The fully adjusted model included 1,247 windows because Kt/V was missing for one window.

**Table S3.** Sensitivity model replacing ΔlogAlb with ΔGNRI (albumin excluded) for concurrent 3-month changes in ΔlogDW (rolling 3-month windows).

Patient-clustered robust standard errors were used. ΔlogX denotes log[X_end/X_start] within each 3-month window.

| **Predictor** | **Unstandardized β** | **Standardized β** | **P value** |
| --- | --- | --- | --- |
| ΔlogBChE | 0.015 | 0.082 | 0.049 |
| ΔloghANP | 0.004 | 0.131 | 0.002 |
| ΔlogCRP | 0.000 | 0.012 | 0.65 |
| ΔGNRI | 0.000 | 0.025 | 0.66 |
| Sex | 0.000 | 0.012 | 0.82 |
| Age, years | -0.000 | -0.004 | 0.95 |
| Diabetes mellitus | -0.003 | -0.181 | <0.001 |
| Dialysis modality  (HDF vs HD) | 0.003 | 0.122 | 0.052 |
| Dialysis vintage, months | -0.000 | -0.067 | 0.18 |
| Delivered Kt/V (baseline) | 0.009 | 0.205 | <0.001 |

**Table S4.** Sensitivity analyses including dialysis modality and cardiothoracic ratio (CTR).

Patient-clustered robust standard errors were used. Models were fit on overlapping 3-month rolling windows. These models extended the primary multivariable model, which included ΔlogBChE, ΔlogAlb, and ΔloghANP, by additionally including dialysis modality, with or without CTR. β coefficients are unstandardized regression coefficients. ΔlogX denotes log[X_end/X_start] within each 3-month window.

| Model | ΔlogBChE β (95% CI) | ΔlogBChE  P value | CTR β  (P value) | HDF β  (P value) | N |
| --- | --- | --- | --- | --- | --- |
| Primary model + dialysis modality | 0.046 (0.010 – 0.083) | 0.013 | — | 0.006  (P = 0.041) | 1247 |
| Primary model + dialysis modality + CTR | 0.041 (0.005 – 0.077) | 0.026 | -0.000453  (P = 0.068) | 0.006  (P = 0.054) | 1231 |

Models included the predictors used in the primary multivariable model, with additional adjustment for dialysis modality, with or without adding CTR. Observation numbers differed across models because of missing data for some covariates, including Kt/V and CTR.

**Table S5.** Sensitivity analyses accounting for within-patient dependence across overlapping 3-month rolling windows.

Outcome and predictors were standardized (z-scores) across all windows; coefficients therefore represent standardized β. ΔlogX denotes log[X_end/X_start] within each 3-month window before standardization.

| Predictor | OLS  (patient-clustered SE) | Linear mixed-effects (random intercept) | GEE (exchangeable working correlation) |
| --- | --- | --- | --- |
| ΔlogBChE | 0.115 (0.041),  P = 0.005 | 0.052 (0.031),  P = 0.096 | 0.052 (0.043),  P = 0.23 |
| ΔlogAlb | -0.036 (0.042),  P = 0.39 | -0.012 (0.029),  P = 0.67 | -0.012 (0.039),  P = 0.75 |
| ΔloghANP | 0.144 (0.045),  P = 0.001 | 0.144 (0.026),  P <0.001 | 0.144 (0.036),  P <0.001 |

Abbreviations: OLS, ordinary least squares; SE, standard error; GEE, generalized estimating equations.

**Table S6.** Variance inflation factors for predictors in the primary multivariable model

| Variable | VIF |
| --- | --- |
| ΔlogBChE | 1.493 |
| ΔlogAlb | 1.363 |
| ΔloghANP | 1.115 |

VIFs were calculated for the primary multivariable model including ΔlogBChE, ΔlogAlb, and ΔloghANP as predictors of ΔlogDW.

**Table S7.** Exploratory analyses of associations between 12-month changes in serum BChE and blood pressure

Panel A. Unadjusted correlations between 12-month changes

| Variables | N | Pearson r | P value | Spearman ρ | P value |
| --- | --- | --- | --- | --- | --- |
| ΔBChE vs ΔDW | 96 | 0.301 | 0.003 | 0.252 | 0.013 |
| ΔBChE vs ΔSBP | 96 | 0.028 | 0.784 | 0.003 | 0.973 |
| ΔBChE vs ΔDBP | 96 | −0.042 | 0.681 | −0.126 | 0.222 |

Panel B. Primary adjusted models

| Outcome | Adjustment variables | ΔBChE coefficient per 50-U/L increase | Robust SE | 95% CI | P value |
| --- | --- | --- | --- | --- | --- |
| ΔSBP | Change in antihypertensive tablet count, ΔDW | 2.239 mmHg | 3.288 | −4.29 to 8.77 | 0.498 |
| ΔDBP | Change in antihypertensive tablet count, ΔDW | −0.167 mmHg | 1.899 | −3.94 to 3.60 | 0.930 |

Panel C. Sensitivity analyses additionally adjusting for baseline BP

| Outcome | Adjustment variables | ΔBChE coefficient per 50-U/L increase | Robust SE | 95% CI | P value |
| --- | --- | --- | --- | --- | --- |
| ΔSBP | Change in antihypertensive tablet count, ΔDW, baseline SBP | 0.512 mmHg | 3.383 | −6.21 to 7.23 | 0.880 |
| ΔDBP | Change in antihypertensive tablet count, ΔDW, baseline DBP | −0.759 mmHg | 1.783 | −4.30 to 2.78 | 0.671 |

Panel D. Sensitivity analyses using percentage change in DW and additionally adjusting for baseline BP

| Outcome | Adjustment variables | ΔBChE coefficient per 50-U/L increase | Robust SE | 95% CI | P value |
| --- | --- | --- | --- | --- | --- |
| ΔSBP | Change in antihypertensive tablet count, %ΔDW, baseline SBP | 0.280 mmHg | 3.245 | −6.17 to 6.73 | 0.931 |
| ΔDBP | Change in antihypertensive tablet count, %ΔDW, baseline DBP | −0.783 mmHg | 1.757 | −4.27 to 2.71 | 0.657 |

Twelve-month changes were calculated as the second annual December assessment minus the first annual December assessment. The number of antihypertensive tablets represents tablet count per day rather than drug class or dose-equivalent treatment intensity. All adjusted models used HC3 heteroskedasticity-robust standard errors. Given the exploratory nature of these analyses, P values were not adjusted for multiple comparisons. %ΔDW was calculated as 100 × (DW at the second assessment − DW at the first assessment) / DW at the first assessment. N = 96 for all analyses.

Abbreviations: BP, blood pressure; SBP, systolic blood pressure; DBP, diastolic blood pressure; SE, standard error

**Table S8.** Correlation between concurrent 3-month changes in biomarkers across rolling windows

| Variables | N (windows) | Spearman ρ | 95% CI  (cluster bootstrap) | P value |
| --- | --- | --- | --- | --- |
| ΔlogBChE vs ΔloghANP | 1248 | -0.315 | -0.371 to -0.260 | <0.001 |

Δlog indicates the difference in natural log-transformed values between the start and end of each 3-month window (log-ratio). The 95% confidence interval was obtained using patient-level cluster bootstrap resampling (2,000 replicates).
